# *urg1:* A Uracil-Regulatable Promoter System for Fission Yeast with Short Induction and Repression Times

**DOI:** 10.1371/journal.pone.0001428

**Published:** 2008-01-16

**Authors:** Stephen Watt, Juan Mata, Luis López-Maury, Samuel Marguerat, Gavin Burns, Jürg Bähler

**Affiliations:** Cancer Research United Kingdom Fission Yeast Functional Genomics Group, Wellcome Trust Sanger Institute, Hinxton, Cambridge, United Kingdom; Victor Chang Cardiac Research Institute, Australia

## Abstract

**Background:**

The fission yeast *Schizosaccharomyces pombe* is a popular genetic model organism with powerful experimental tools. The thiamine-regulatable *nmt1* promoter and derivatives, which take >15 hours for full induction, are most commonly used for controlled expression of ectopic genes. Given the short cell cycle of fission yeast, however, a promoter system that can be rapidly regulated, similar to the *GAL* system for budding yeast, would provide a key advantage for many experiments.

**Methodology/Principal Findings:**

We used *S. pombe* microarrays to identify three neighbouring genes (*urg1, urg2,* and *urg3*) whose transcript levels rapidly and strongly increased in response to uracil, a condition which otherwise had little effect on global gene expression. We cloned the promoter of *urg1* (uracil-regulatable gene) to create several PCR-based gene targeting modules for replacing native promoters with the *urg1* promoter (P*urg1*) in the normal chromosomal locations of genes of interest. The *kanMX6* and *natMX6* markers allow selection under *urg1* induced and repressed conditions, respectively. Some modules also allow N-terminal tagging of gene products placed under *urg1* control. Using *pom1* as a proof-of-principle, we observed a maximal increase of P*urg1-pom1* transcripts after uracil addition within less than 30 minutes, and a similarly rapid decrease after uracil removal. The induced and repressed transcriptional states remained stable over 24-hour periods. RT-PCR comparisons showed that both induced and repressed P*urg1-pom1* transcript levels were lower than corresponding P3*nmt1-pom1* levels (wild-type *nmt1* promoter) but higher than P81*nmt1-pom1* levels (weak *nmt1* derivative).

**Conclusions/Significance:**

We exploited the *urg1* promoter system to rapidly induce *pom1* expression at defined cell-cycle stages, showing that ectopic *pom1* expression leads to cell branching in G2-phase but much less so in G1-phase. The high temporal resolution provided by the *urg1* promoter should facilitate experimental design and improve the genetic toolbox for the fission yeast community.

## Introduction

The experimental manipulation of expression levels from specific genes is a key genetic approach to elucidate gene function in model organisms. A range of regulatable promoter systems have been described for use in the fission yeast, *Schizosaccharomyces pombe* [1], [2]. The most widely applied system is based on the thiamine-repressible *nmt1* promoter and two weakened derivatives [3]–[5]. While these promoters offer a wide choice of transcription levels, they take ∼15–21 hours to reach maximum induction once thiamine is removed from the medium, and ∼2–4 hours for full repression after thiamine addition. A truncated version of the *nmt1* promoter shows altered characteristics [6]: it reaches maximum expression within 3 hours but requires a temperature shift for induction, which is expected to trigger a cellular stress response [7].

Other promoter systems have been described for fission yeast. One system is based on *ctr4* [8], which is strongly induced within 3 hours in the absence of copper. The addition of a copper chelator as an inducing agent, however, leads to a large transcriptional response [9]. The *inv1* promoter fully induces transcription within one hour in the absence of glucose in sucrose-based medium, but induction is only transient as sucrose is hydrolyzed to glucose, leading to *inv1* repression after ∼2 hours [10]. The *fbp1* promoter is also glucose-repressible [11]. Changes in carbon sources, however, lead to substantial transcriptional and metabolic responses ([12]; LLM and JB, unpublished observation). The *hsp16* promoter is activated within a few hours by heat shock or other stresses [13], conditions that will also trigger substantial stress responses [7]. The ectopic *CaMV* promoter is induced by tetracycline [14] or by anhydrotetracycline that is a superior inducing agent [15]. This promoter is regulatable in both minimal and rich media and is fully induced within 12 and 9 hours in the two media types, respectively [15]. The *CaMV35S* promoter shows low basal expression levels under repressed conditions [15], which should make it useful to study essential proteins.

Since the fission yeast cell cycle is completed within 2–3 hours, the lack of a promoter system that can be rapidly regulated, similar to the *GAL* system for budding yeast [16], is a serious drawback for many experiments. We used genome-wide expression data to identify conditions that lead to a strong and rapid regulation of few specific genes. This approach culminated in the development of the uracil-regulatable *urg1* promoter system, which allows tight expression control of ectopic genes with otherwise minimal side effects on genome-wide gene expression.

## Results and Discussion

**Figure 1:**
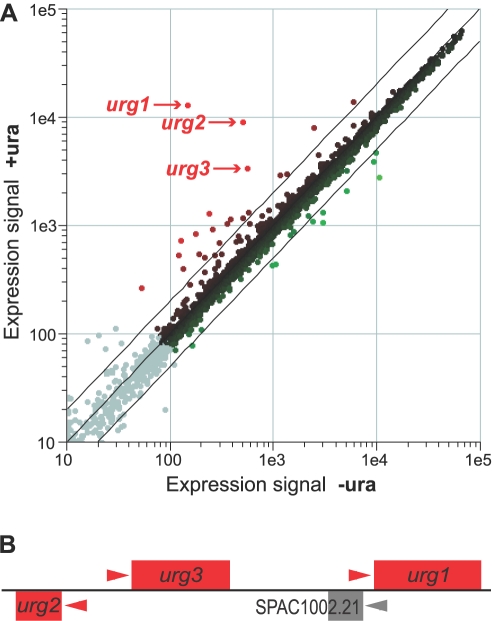
The three *urg* genes are induced with uracil and clustered in genome. (A) Scatter plot showing microarray signal intensities for transcripts from cells grown in the presence (Y-axis) or absence (X-axis) of uracil, whose transcriptomes were competitively hybridized on the same microarray. The *urg1, urg2,* and *urg3* genes are most strongly induced in response to uracil, a condition that otherwise triggers only minor gene expression changes. The grey dots reflect transcripts that were flagged ‘absent’ during initial data processing [37]. (B) Genomic arrangement of *urg1, urg2,* and *urg3* genes on chromosome I. Arrowheads indicate transcriptional direction. The SPAC1002.21 open reading frame between *urg1* and *urg3* may not be a real gene: it is annotated as ‘dubious’ in *S. pombe* GeneDB and does not seem to be expressed in any conditions based on microarray data (unpublished observations).

Microarrays are ideal to screen for genes that are distinctly regulated under selected conditions that otherwise have little effect on global gene expression. In a study to determine how commonly used media supplements affected global transcriptional patterns in fission yeast, we identified the three ‘uracil-regulatable genes’ *urg1* (SPAC1002.19), *urg2* (SPAC1002.17c), and *urg3* (SPAC1002.18) whose transcript levels were highly increased when the pyrimidine base uracil was present in the medium, a condition that affected the expression of only ∼0.5% of all genes (Figure 1A). This effect was not transient: the increased transcript levels were maintained after 24 hours in the continued presence of uracil (data not shown). Notably, the three *urg* genes were clustered together on chromosome I (Figure 1B). *urg1* encodes a protein of the GTP cyclohydrolase II family of enzymes involved in riboflavin biosynthesis [17]. *urg2* encodes a protein similar to the budding yeast Fur1p uracil phosphoribosyltransferase of the pyrimidine salvage pathway [18], while *urg3* encodes a protein of unknown function with a DUF1688 domain.

**Figure 2:**
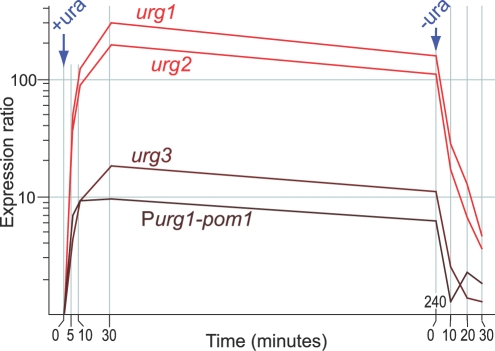
The *urg* promoters control changes in mRNA levels in response to uracil. Timecourse experiment showing the gene expression profiles of *urg1, urg2, urg3,* and P*urg1-pom1* at 5, 10, 30, and 240 minutes after uracil addition, and at 10, 20, and 30 minutes after uracil removal. The Y-axis shows gene expression ratios relative to the same cells grown without uracil (0 minute timepoint). Gene expression ratios were determined using microarrays. Note that the presence of a second *urg1* promoter in the same cells (P*urg1-pom1*) did not affect the expression characteristics of the *urg1* gene.

To analyse the regulation of the *urg* genes in more detail, we determined their expression profiles at different times after addition and removal of uracil (Figure 2). All three genes were rapidly induced in uracil-containing medium, showing highly increased transcript levels after 5 minutes of uracil addition and peaking in transcript levels within 30 minutes. Similarly, transcript levels of all three genes rapidly dropped after transfer to medium without uracil. These results encouraged us to develop a new promoter system for the rapid and specific regulation of ectopic genes. We focussed on *urg1* as it is the most strongly regulated gene in response to uracil (Figure 1A; Figure 2). The difference in relative regulation between *urg1, urg2,* and *urg3* seems to mainly reflect differences in basal expression levels: while all three genes show similar absolute expression signals in rich medium (containing uracil), the expression signals in minimal medium are ∼3- and 4-fold higher for *urg2* and *urg3*, respectively, compared to *urg1* based on Affymetrix chip data [19]. Cells deleted for *urg1* were viable and showed wild-type growth rates, both in the presence and absence of uracil (data not shown).

We analyzed available microarray data for *urg1* expression patterns under different conditions. In vegetative cells growing in the absence of uracil, *urg1* shows close to background signals on microarrays and is among the 10% most lowly expressed genes [19], and it is marginally periodically expressed during the cell cycle [20]. The *urg1* transcripts were induced ∼10- to 20-fold in response to cadmium and *t*-butylhydroperoxide but not in response to heat shock, sorbitol, MMS, H_2_O_2_, or menadione [7], [21]. The *urg1* transcripts were also induced ∼10- to 20-fold in late meiosis and, most strongly, during nitrogen starvation, where transcript levels increased >100-fold [22]–[24], which is similar to the response in uracil described above. It is possible that *urg1* is involved in recycling uracil as an alternative nitrogen source. Interestingly, *urg1* was even more highly expressed in an *ura4* deletion background than in a wild-type background in the presence of uracil, and conversely, *ura4* was more highly expressed in an *urg1* deletion background (unpublished microarray data). Thiamine, which represses the *nmt1* promoter, has no influence on *urg1* expression levels ([25]; and unpublished data).

To develop a new regulatable promoter system, we cloned *urg1* promoter fragments of different sizes (232, 675, and 924 bp upstream of *urg1* start codon) into the pFA6a-kanMX6-P3nmt1 module [26], replacing the *nmt1* with *urg1* promoter fragments. We then applied PCR-based gene targeting with these three new cassettes to put the *pom1* gene [27] under the control of the *urg1* promoter fragments. The 232 bp fragment showed constitutively active transcription, whereas the 675 and 924 bp fragments both led to similarly regulated transcription in response to uracil (data not shown). These data suggest that the first 232 bp upstream of the ATG start codon are sufficient for active transcription whereas the sequences between 232 and 675 bp are required to down-regulate transcription in the absence of uracil.

**Figure 3:**
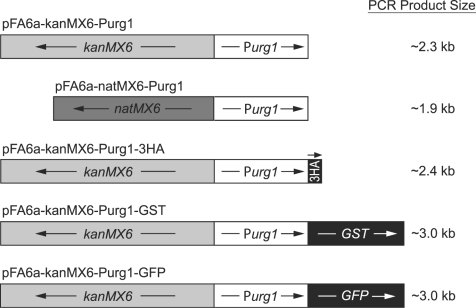
Modules for PCR-based gene targeting to place genes under P*urg1* control and N-terminal tagging of proteins. These modules are derived from previously published modules [26], [28] using a 675 bp fragment immediately upstream of the *urg1* open reading frame. Transcriptional directions are indicated by arrows. Restriction sites and tags are as described before (Figure 2 in [26]); the GFP tag carries the S56T mutation [39]. The approximate sizes of the expected PCR products are indicated at right.

Based on these data, we cloned the 675 bp fragment containing the functional *urg1* promoter (P*urg1*) into several PCR-based targeting vectors for straightforward integration of the promoter upstream of selected genes in their normal chromosomal locations (Figure 3). The available modules contain the kanMX6 or natMX6 dominant markers, allowing selection for cells resistant to the antibiotics G418 or nourseothricin (NAT), respectively [26], [28]. NAT allows easier selection on minimal medium (without uracil), which can be advantageous in situations where constructs with the active *urg1* promoter lead to sick or dead cells. If required, the products of genes placed under P*urg1* control can also be N-terminally tagged with 3HA, GST, or GFP_(S65T)_ [26]. All the modules shown in Figure 3 can be amplified using the same forward primer, but they require different reverse primers (Table 1). Genomic integration of P*urg1* ensures more controlled and homogeneous expression levels compared to analyses involving multi-copy plasmids, which show great variations in copy number. The 675-bp P*urg1* fragment was used in all experiments below.

**Table 1:** PCR primers for amplification of the modules in Figure 3.

Module	Primer sequence
All modules (*forward*)^1^	5′-(gene-specific sequence)-GAATTCGAGCTCGTTTAAAC-3′
pFA6a-kanMX6-Purg1 (*reverse*)^2^	5′-(gene-specific sequence)-*CAT*ATTGAATTAGTTCTAATTTAGT-3′
pFA6a-natMX6-Purg1 (*reverse*)^2^	5′-(gene-specific sequence)-*CAT*ATTGAATTAGTTCTAATTTAGT-3′
pFA6a-kanMX6-Purg1-3HA (*reverse*)^3^	5′-(gene-specific sequence)-GCA CTG AGC AGC GTA ATC TG-3′
pFA6a-kanMX6-Purg1-GST (*reverse*)^3^	5′-(gene-specific sequence)-ACG CGG AAC CAG ATC CGA TT-3′
pFA6a-kanMX6-Purg1-GFP (*reverse*)^3^	5′-(gene-specific sequence)-TTT GTA TAG TTC ATC CAT GC-3′

1 The forward primer is identical for all modules described here and is the same as for previously described modules containing *nmt1-*derived promoters [26]. The gene-specific portion of the primer is typically chosen to correspond to sequences 100–200 bp upstream of the start codon. A web-based tool for automated primer design is available for these primers [35].

2 A 25-mer universal sequence is used to anneal to P*urg1* due to the AT-rich nature of this sequence; the gene-specific portion is therefore reduced to 75 bp for 100-mer primers, which does not seem to affect targeting efficiency. The complement start codon is indicated in *italic*. For regulated expression of full length proteins, the gene-specific portion of the primer corresponds to the complement of the N-terminal codons of the target gene (*without* start codon).

3 The reading frames of the tag sequences are indicated. These primers are the same as for the corresponding modules containing *nmt1-*derived promoters [26]. For N-terminal tagging of full-length proteins, the gene-specific portion of the primer corresponds to the complement of the N-terminal codons of the target gene (*including* start codon). Note that the 3′ portions of these primers are specific to the tags and correspond to the complement of the C-terminal tag codons (without stop codon). A web-based tool for automated primer design is available for these primers [35].

**Figure 4:**
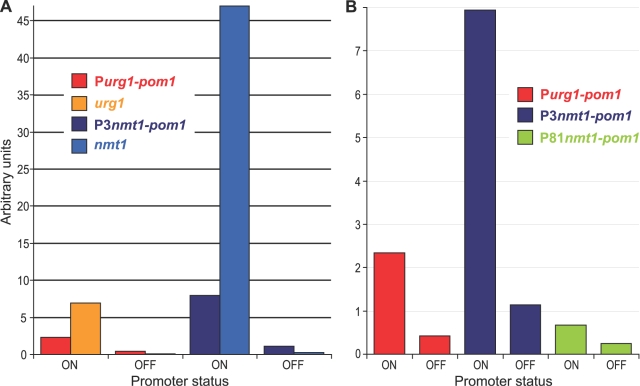
Quantitative comparison of regulation by *urg1* and *nmt1* promoters using *pom1* as reporter. (A) Histogram showing mRNA expression levels determined by qRT-PCR for *pom1* under control of P*urg1* and P3*nmt1* compared to expression levels of *urg1* and *nmt1* genes themselves (colour-coded as indicated in the Figure). Expression levels were determined under both induced (ON) and repressed (OFF) conditions for the two promoter systems. (B) Histogram as in (A) comparing mRNA expression levels of *pom1* under control of P*urg1,* P3*nmt1,* and P81*nmt1.* The same arbitrary units are used for (A) and (B). Cells were grown for two hours either in the presence or absence of uracil (for P*urg1*), or for 21 hours in the presence or absence of thiamine (for P3*nmt1* and P81*nmt1*).

We tested the *urg1* promoter system by placing *pom1* under the control of P*urg1* using the pFA6a-kanMX6-Purg1 cassette (Figure 3; Table 2, strain JB381). The expression profile of *pom1* driven by the *urg1* promoter (P*urg1-pom1*) closely reflected the profiles of the *urg* genes, showing similar timing of induction and repression upon addition and removal of uracil, respectively (Figure 2). Maximal P*urg1-pom1* induction was reached within 10 minutes, which was ∼2.2-fold higher than *pom1* expression levels driven from its own promoter (based on microarray data). Rapid induction and repression time of P*urg1-pom1,* similar to *urg1* under its own promoter, are also evident from PCR-based assays reported before (Figure S9 in [19]). The ∼10-fold induction of *pom1,* however, was lower than for the *urg* genes themselves. Several factors could contribute to this difference in relative regulation. Some of this difference is due to higher basal *pom1* expression: qRT-PCR data showed that P*urg1-pom1* is ∼3.8-fold higher expressed than *urg1* in the absence of uracil (Figure 4A). Moreover, changes in half-live of transcripts can affect relative transcript changes; the 3′-untranslated region (UTR) of *urg1* contains an AU-rich element (ARE) consensus sequence [29], consistent with posttranscriptional control contributing to strong changes in mRNA levels [30], [31]. It is possible that inserting the 3′-UTR of *urg1* behind genes already under the control of P*urg1* would support lower basal transcript levels and larger relative transcript changes after uracil addition. Alternatively or in addition, the genomic context could influence the low expression of *urg1* genes under repressed conditions. Consistent with this possibility, the expression levels of *urg1* and *urg2* increase in several silencing mutants [32], suggesting that this genomic region is relatively silent.

**Table 2:** Strains used in this study.

Strain	Genotype	Source
JB22	*972 h^−^*	Lab collection
JB151	*kanMX6-*P3*nmt1-pom1 h^−^*	[34]
JB178	*kanMX6-*P81*nmt1-3HA-pom1 h^−^*	Lab collection
JB381	*kanMX6-*P*urg1-pom1 h^−^*	This study
JB383	*urg1Δ::kanMX6 h^−^*	This study
JB506	*cdc10-V50 leu1-32 h^+^*	Lab collection
JB508	*cdc25-22 ura4-D18 h^+^*	Lab collection
JB509	*cdc10-V50 kanMX6-*P*urg1-pom1*	This study
JB511	*cdc25-22 kanMX6-*P*urg1-pom1*	This study

To compare the quantitative regulation by P*urg1* with two widely used *nmt1-*based promoters (the strong P3*nmt1* and weak P81*nmt1*; [3], [5]), we performed qRT-PCR analysis of *pom1* under control of these three ectopic promoter systems (Table 2, strains JB381, JB151, and JB178). Figure 4A compares the regulation of *pom1* under control of either P*urg1* or P3*nmt1* with the regulation of *urg1* and *nmt1* under control of their native promoters. Both *urg1* and *nmt1* are more tightly regulated (higher induced and lower repressed transcript levels) than P*urg1-pom1* and P3*nmt1-pom1.* The *nmt1* gene under activating conditions is among the most highly expressed genes in the *S. pombe* genome [19], [33], and expression levels driven by P*urg1* are arguably closer to physiological levels for most genes. Note that the relative mRNA levels for ectopic genes put under control of regulatable promoters will be strongly affected by features such as chromatin context and mRNA stability, and different genes may show different regulation. Figure 4B compares the regulation of *pom1* under control of P*urg1,* P3*nmt1,* or P81*nmt1.* Under induced conditions, P*urg1-pom1* was ∼3.5-fold more expressed than P81*nmt-pom1* but ∼3.5-fold lower expressed than P3*nmt1-pom1.* Under repressed conditions, P*urg1-pom1* was ∼1.7-fold more expressed than P81*nmt1-pom1* but ∼2.7-fold lower expressed than P3*nmt1-pom1.* These data are consistent with gene expression ratios observed after competitively hybridizing samples to DNA microarrays (induced conditions, P*urg1-pom1/*P3*nmt1-pom1*: 2.9, P*urg1-pom1/*P81*nmt1-pom1*: 0.3; repressed conditions: P*urg1-pom1/*P3*nmt1-pom1*: 1.6, P*urg1-pom1/*P81*nmt1-pom1*: 0.6). Our *nmt1* data are also similar to data from a previous comparative analysis [1]. We conclude that for both induced and repressed conditions, the gene expression levels attained from P*urg1* are between those attained from P81*nmt1* and P3*nmt1*.

**Figure 5:**
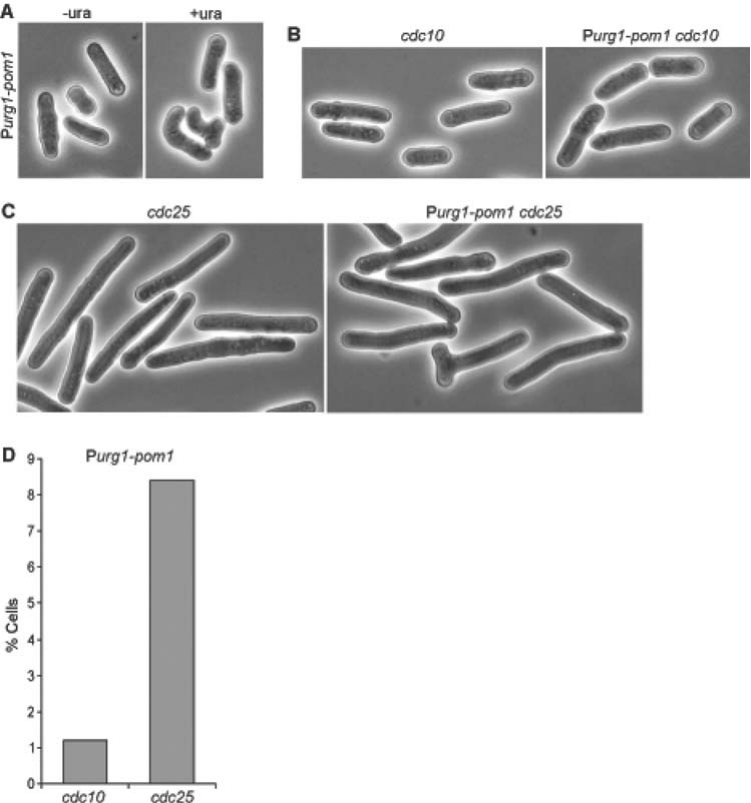
Expression of *pom1* under control of P*urg1* leads to cell branching in G2-phase. (A) P*urg1-pom1* cells were gown without uracil (left); uracil was then added to the same culture and cells were grown for another two hours (right). Cells show no morphological aberrations under repressed conditions but form misplaced growth sites after activation of P*urg1.* (B) *cdc10* and *cdc10* P*urg1-pom1* cells grown for two hours at restrictive temperature without uracil and two hours at restrictive temperature with uracil. Activation of P*urg1* has little effect on cell morphology. (C) *cdc25* and *cdc25* P*urg1-pom1* cells grown for two hours at restrictive temperature without uracil and two hours at restrictive temperature with uracil. Activation of P*urg1* leads to misplaced growth sites. (D) Histogram comparing percentage of branched cells when P*urg1-pom1* is activated in either *cdc10* or *cdc25* backgrounds.

These findings are corroborated by phenotype data. Deletion of *pom1* leads to aberrantly positioned polarized growth and cytokinesis [27], while overexpression of *pom1* under the control of P3*nmt1* leads to branched cells after 19 hours and to depolarized, round cells within 25 hours [34]. In the absence of uracil, the P*urg1-pom1* cells looked like wild-type cells (Figure 5A), suggesting that basal transcription from the *urg1* promoter provides sufficient *pom1* expression to prevent the defects associated with *pom1* mutants. The *pom1* expression levels are ∼3.8-fold below average transcript levels [19], and repressed P*urg1-pom1* levels were ∼90% of the native *pom1* levels (based on microarray data). We conclude that the basal expression level from P*urg1* can be sufficient to fully support gene function, at least for relatively lowly expressed genes such as *pom1.*

Induction of P*urg1-pom1* expression by uracil addition led to increasing numbers of bent and branched cells already after two hours (Figure 5A), although these cells did not become round even after 25 hours of P*urg1-pom1* expression, consistent with weaker *pom1* expression than in P3*nmt1-pom1* cells. In contrast, P81*nmt1-pom1* cells did not show any cell branching even 25 hours after uracil addition, consistent with weaker *pom1* expression than in P*urg1-pom1* cells. These findings are consistent with expression levels driven by P*urg1* being between those of P81*nmt1* and P3*nmt1,* and they illustrate the dramatic decrease in timing of transcriptional induction when using P*urg1*.

As a further proof-of-principle, we used the P*urg1-pom1* cells to perform a cell-cycle experiment that would be difficult with the *nmt1* promoter. Available data suggest that Pom1p can activate growth during the G2-phase of the cell cycle: 1) cells deleted for *pom1* cannot activate a second growth site at the new end and thus fail to initiate bipolar growth during G2-phase [27]; 2) Pom1p kinase activity is cell-cycle regulated and is higher in G2-phase than in G1-phase [34]; and 3) overexpression of *pom1* leads to branched cells, indicating mislocalized growth sites (see above). Taking these data together, we would predict that Pom1p can promote cell branching when overexpressed in G2-phase but not when overexpressed in G1-phase. To test this hypothesis, we combined P*urg1-pom1* with the temperature-sensitive *cdc10* and *cdc25* mutants, which arrest in G1- and G2-phases, respectively, at the restrictive temperature of 36°C (Table 2, strains JB509 and JB511). We incubated the *cdc10* P*urg1-pom1* and *cdc25* P*urg1-pom1* strains at 36°C to enrich for cells in G1- and G2-phases, respectively. After two hours, we added uracil to the medium to induce *pom1* expression and incubated the cells for another two hours at 36°C. As predicted, the *cdc25* P*urg1-pom1* strain showed an about 7-fold higher proportion of branched cells than the *cdc10* P*urg1-pom1* strain (Figure 5B–D). It is possible that the few branched cells in the *cdc10* background reflect that two hours at 36°C was not sufficient to completely arrest all cells in G1-phase. These data support the notion that Pom1p can activate growth during the G2- but not during the G1-phase of the cell cycle. Note that this type of experiment would be very complicated or impossible with the *nmt1* promoter system due to the long induction times.

### Conclusion

We believe that the *urg1* system will prove to be a popular and valuable addition to the genetic toolbox available to fission yeast researchers. Besides regulation of the three *urg* genes, clustered together on chromosome I, the addition of uracil has only minimal effects on global gene expression and should affect cellular physiology less than the changes in carbon sources required for the budding yeast *GAL* promoter system. This specific effect on gene expression will also make it easier to interpret regulatory effects of genes under *urg1* promoter control in genome-wide studies. The *urg1* promoter system could also be used to control gene expression in specialized situations, such as to induce ectopic genes in nitrogen-starved cells, a condition that leads to *urg1* induction in the absence of uracil. The *pom1* gene under P*urg1* control is fully induced and repressed within ∼10 minutes of uracil addition and removal, respectively. Both induced and repressed P*urg1-pom1* transcript levels are intermediate between those from the weakest and the strongest *nmt1* promoter driving *pom1*. Probably, P*urg1* will be most useful to rapidly induce transcription of selected genes, e.g. to provide a pulse of expression during a defined cell-cycle stage. As most promoters, P*urg1* supports substantial basal expression levels even when ‘switched off’. As for the *nmt1* promoter, regulation of *urg1* transcripts themselves is tighter and stronger compared to the regulation of ectopic transcripts by P*urg1*. This difference could reflect local chromatin environment and/or additional posttranscriptional control. In any case, the half-lives of different transcripts will affect changes in transcript levels, and addition of the 3′-UTR of *urg1* might promote a tighter regulation of ectopic transcripts. Future refinements of the *urg* promoter system, including manipulations of the promoter sequence and analysis of uracil concentration effects, may further increase its usefulness.

## Materials and Methods

### Strains and yeast experiments

Strains used in this study are listed in Table 2. Strain JB381 was constructed using the new pFA6a-kanMX6-Purg1 module (Figure 3) and transformed as described [26]. 100-mer primers were designed using PPPP [35] such that 160 bp of the native *pom1* promoter were replaced with P*urg1*. Transformed cells were checked for correct integration by colony PCR using a forward primer in P*urg1* (5′-ATAAATAAGGGAGGAAATCCATACG-3′), whose 5′-end is located 203 bp upstream of the ATG start codon of *urg1,* and a reverse primer complementary to *pom1*. Strain JB383 was created by PCR-based gene deletion [26]. Strains JB509 and JB511 were created by crossing JB381 with JB506 and JB508.

Cells were grown at 32°C in Edinburgh Minimal Medium (EMM) [36], adding either uracil at 0.25 mg/ml to induce P*urg1*, or 15 µM thiamine to repress the *nmt1* promoter. For the experiment in Figure 2, JB381 cells were grown to ∼5×10^6^ cells/ml before uracil addition; after four hours, cells were filtered, washed once in 32°C EMM without uracil, and incubated in 32°C EMM without uracil for another 30 minutes. For Figure 5A, JB381 cells were grown in EMM without uracil, before adding uracil and growing for two hours. For the experiment in Figure 5B–D, JB506, JB508, JB509 and JB511 cells were grown at 25°C to ∼5×10^6^ cells/ml, shifted to 36°C and grown for two hours, at which time uracil was added, and grown for another two hours at 36°C.

### Construction of P*urg1* modules

To construct the modules of Figure 3, the *urg1* promoter (P*urg1*) was amplified from *S. pombe* genomic DNA by PCR using the following primers: urg1F675 (5′-AAAAGATCTCGATTAGCGTGACACGGATT-3′) and urg1R (5′-AAATTAATTAACCTTTGTTCAGTGGCAAGCAT-3′) containing *Bgl*II and *Pac*I sites (underlined) for cloning into the corresponding sites of the pFA6a-MX6 vectors [26], [28]. For the smaller and larger *urg1* promoter fragments tested, we used the following two forward primers instead: urg1F232 (5′-AAAAGATCTGCGCTTTCATTGATAGTATCTG-3′), urg1F924 (5′-AAAAGATCTTGCACTCAGCGTAAAGTCAAG-3′). All PCR amplifications were carried out with HiFi-Platinum Taq (Invitrogen), and ligations were carried out using T4 DNA ligase (Roche) and transformed into DH5α competent cells (Invitrogen). Plasmid DNA was extracted using the Illustra GFX Micro Plasmid kit (GE Healthcare).

### Microscopy

Light microscopy was carried out using a Carl Zeiss Axiostar equipped with a Canon Powershot A95 camera. Images were captured with the Canon ZoomBrowser EX software. For Figure 5, at least 200 cells were counted.

### Microarray analysis

RNA was extracted and processed for microarray hybridization as described before ([37]; www.sanger.ac.uk/PostGenomics/S_pombe). Cy3 and Cy5 (GE Healthcare) incorporation was carried out using the Invitrogen Superscript direct cDNA labelling system according to manufacturer's instructions. For the timecourse experiment in Figure 2, all timepoints were pooled and used as a common reference for each timepoint. Microarrays were scanned using an Axon GenePix 4000B scanner and analyzed with GenePix 6.0 software. Quality control and data normalization was carried out as described [37]. Results were visualized with GeneSpring GX 7.3 (Agilent). The processed microarray data are available from our website: www.sanger.ac.uk/PostGenomics/S_pombe.

### Quantitative RT-PCR

For the qRT-PCR experiment in Figure 4, cells were grown for two hours either in the presence or absence of uracil (strain JB381), or for 21 hours in the presence or absence of thiamine (strains JB151 and JB178). RNA was isolated and purified as described [37] and treated with Turbo DNA-free (Ambion). Reverse transcription reactions were performed using Superscript III (Invitrogen). qRT-PCR reactions were carried out using Taqman specific probes (Sigma) and Platinum qPCR mix with ROX (Invitrogen) on an Applied Biosystem 7900HT system according to manufacturer's instructions. All primers and probes were designed using Primer3 software ([38]; http://primer3.sourceforge.net). Expression levels for *urg1, nmt1,* and *pom1* were determined from two repeats against a standard curve. Arbitrary expression units were calculated using a standard curve for each probe and primer set from serial dilutions of *S. pombe* genomic DNA. The following fluorescent probes with 6-FAM as 5′-end reporter and TAMRA as 3′-end quencher were used: P-C1223.02: 5′-TTATTCCAAGCGTTTGGGCATCATC-3′; P-C2F7.03c: 5′-CCTTTACCGAATTTGCCAATGGAAT-3′; P-C1002.19: 5′-CATTAAGAAGATTGACCGCATGCTCA-3′; P-C19C2.07: 5′-TACTTCTCCATTGCCGCCGCTTT-3′; and P-C1322.04: 5′-TGGTGACGTTAATATTGGTCGCAATG-3′. The following PCR primers were used: Q-C1223.02F: 5′-TCCCCAGAGATTGGAACAAG-3′; Q-C1223.02R: 5′-TTCTCATCGGGGTCAAGTTC-3′; Q-C2F7.03cF: 5′-TGCGAGACCCCCAAATATAG-3′; Q-C2F7.03cR: 5′-CTCTTTCGGGGAAGGTAAGG-3′; Q-C1002.19F: 5′-GCGTTTCCAAGCTCTTATGC-3′; Q-C1002.19R: 5′-AACAATGGCATCATGCTTCA-3′; Q-C19C2.07F: 5′-CGTGAGCTCTCCTCCGTTAC-3′; Q-C19C2.07R: 5′-TTACCGGGCTTGTAGACACC-3′; Q-C1322.04F: TTCCCAGCATTCCAAAAATC-3′; Q-C1322.04R: 5′-GTTGGCATCACTAGCGACAA-3′.

## References

[1] Forsburg SL (1993). Comparison of *Schizosaccharomyces pombe* expression systems.. Nucleic Acids Res.

[2] Siam R, Dolan WP, Forsburg SL (2004). Choosing and using *Schizosaccharomyces pombe* plasmids.. Methods.

[3] Basi G, Schmid E, Maundrell K (1993). TATA box mutations in the *Schizosaccharomyces pombe nmt1* promoter affect transcription efficiency but not the transcription start point or thiamine repressibility.. Gene.

[4] Maundrell K (1990). *nmt1* of fission yeast. A highly transcribed gene completely repressed by thiamine.. J Biol Chem.

[5] Maundrell K (1993). Thiamine-repressible expression vectors pREP and pRIP for fission yeast.. Gene.

[6] Kumar R, Singh J (2006). A truncated derivative of nmt 1 promoter exhibits temperature-dependent induction of gene expression in *Schizosaccharomyces pombe*.. Yeast.

[7] Chen D, Toone WM, Mata J, Lyne R, Burns G (2003). Global transcriptional responses of fission yeast to environmental stress.. Mol Biol Cell.

[8] Bellemare DR, Sanschagrin M, Beaudoin J, Labbé S (2001). A novel copper-regulated promoter system for expression of heterologous proteins in *Schizosaccharomyces pombe*.. Gene.

[9] Rustici G, van Bakel H, Lackner DH, Holstege FC, Wijmenga C (2007). Global transcriptional responses of fission and budding yeast to changes in copper and iron levels: a comparative study.. Genome Biol.

[10] Iacovoni JS, Russell P, Gaits F (1999). A new inducible protein expression system in fission yeast based on the glucose-repressed *inv1* promoter.. Gene.

[11] Hoffman CS, Winston F (1989). A transcriptionally regulated expression vector for the fission yeast *Schizosaccharomyces pombe*.. Gene.

[12] DeRisi JL, Iyer VR, Brown PO (1997). Exploring the metabolic and genetic control of gene expression on a genomic scale.. Science.

[13] Fujita Y, Tohda H, Giga-Hama Y, Takegawa K (2006). Heat shock-inducible expression vectors for use in *Schizosaccharomyces pombe*.. FEMS Yeast Res.

[14] Faryar K, Gatz C (1992). Construction of a tetracycline-inducible promoter in *Schizosaccharomyces pombe*.. Curr Genet.

[15] Erler A, Maresca M, Fu J, Stewart AF (2006). Recombineering reagents for improved inducible expression and selection marker re-use in *Schizosaccharomyces pombe*.. Yeast.

[16] Johnston M, Davis RW (1984). Sequences that regulate the divergent GAL1-GAL10 promoter in *Saccharomyces cerevisiae*.. Mol Cell Biol.

[17] Ren J, Kotaka M, Lockyer M, Lamb HK, Hawkins AR (2005). GTP cyclohydrolase II structure and mechanism.. J Biol Chem.

[18] Kern L, de Montigny J, Jund R, Lacroute F (1990). The FUR1 gene of *Saccharomyces cerevisiae*: cloning, structure and expression of wild-type and mutant alleles.. Gene.

[19] Lackner DH, Beilharz TH, Marguerat S, Mata J, Watt S (2007). A Network of Multiple Regulatory Layers Shapes Gene Expression in Fission Yeast.. Mol Cell.

[20] Marguerat S, Jensen TS, de Lichtenberg U, Wilhelm BT, Jensen LJ (2006). The more the merrier: comparative analysis of microarray studies on cell cycle-regulated genes in fission yeast.. Yeast.

[21] Chen D, Wilkinson C, Watt S, Penkett C, Toone W (2008). Multiple pathways differentially regulate global oxidative stress responses in fission yeast.. Mol Biol Cell.

[22] Mata J, Bähler J (2006). Global roles of Ste11p, cell type, and pheromone in the control of gene expression during early sexual differentiation in fission yeast.. Proc Natl Acad Sci U S A.

[23] Mata J, Lyne R, Burns G, Bähler J (2002). The transcriptional program of meiosis and sporulation in fission yeast.. Nat Genet.

[24] Mata J, Wilbrey A, Bähler J (2007). Transcriptional regulatory network for sexual differentiation in fission yeast.. Genome Biol.

[25] Jenkins CCL, Mata J, Crane RF, Thomas B, Akoulitchev A (2005). Activation of AP-1-Dependent Transcription by a Truncated Translation Initiation Factor.. Eukaryotic Cell.

[26] Bähler J, Wu JQ, Longtine MS, Shah NG, McKenzie A (1998). Heterologous modules for efficient and versatile PCR-based gene targeting in *Schizosaccharomyces pombe*.. Yeast.

[27] Bähler J, Pringle JR (1998). Pom1p, a fission yeast protein kinase that provides positional information for both polarized growth and cytokinesis.. Genes & Development.

[28] Hentges P, Van Driessche B, Tafforeau L, Vandenhaute J, Carr AM (2005). Three novel antibiotic marker cassettes for gene disruption and marker switching in *Schizosaccharomyces pombe*.. Yeast.

[29] Bakheet T, Williams BR, Khabar KS (2006). ARED 3.0: the large and diverse AU-rich transcriptome.. Nucleic Acids Res.

[30] Garneau NL, Wilusz J, Wilusz CJ (2007). The highways and byways of mRNA decay.. Nat Rev Mol Cell Biol.

[31] Mata J, Marguerat S, Bähler J (2005). Post-transcriptional control of gene expression: a genome-wide perspective.. Trends Biochem Sci.

[32] Hansen KR, Burns G, Mata J, Volpe TA, Martienssen RA (2005). Global effects on gene expression in fission yeast by silencing and RNA interference machineries.. Mol Cell Biol.

[33] Mata J, Bähler J (2003). Correlations between gene expression and gene conservation in fission yeast.. Genome Res.

[34] Bähler J, Nurse P (2001). Fission yeast Pom1p kinase activity is cell cycle regulated and essential for cellular symmetry during growth and division.. EMBO J.

[35] Penkett CJ, Birtle ZE, Bähler J (2006). Simplified primer design for PCR-based gene targeting and microarray primer database: two web tools for fission yeast.. Yeast.

[36] Moreno S, Klar A, Nurse P (1991). Molecular genetic analysis of fission yeast *Schizosaccharomyces pombe*.. Methods Enzymol.

[37] Lyne R, Burns G, Mata J, Penkett CJ, Rustici G (2003). Whole-genome microarrays of fission yeast: characteristics, accuracy, reproducibility, and processing of array data.. BMC Genomics.

[38] Rozen S, Skaletsky H (2000). Primer3 on the WWW for general users and for biologist programmers.. Methods Mol Biol.

[39] Heim R, Cubitt AB, Tsien RY (1995). Improved green fluorescence.. Nature.

